# Heritability of living in deprived neighbourhoods

**DOI:** 10.1038/tp.2016.215

**Published:** 2016-11-08

**Authors:** A Heinz, U Kluge, M A Rapp

**Affiliations:** 1 Department of Psychiatry and Psychotherapy, Charité University Medicine Berlin, Campus Mitte, Berlin, Germany; 2 Social and Preventive Medicine, University of Potsdam, Potsdam, Germany.; 3 These authors contributed equally to this work.

With great interest we read the paper of Sariaslan *et al.*,^1^ who report that their ‘sibling data suggest that living in deprived neighbourhoods was substantially heritable', with genetic factors explaining up to 2/3 of the variance. As this finding has substantial implications for social and health-care policies in inner cities with poor neighbourhoods, we would like to address two points:

First, not reported in the abstract but in the results section, Sariaslan *et al.*^1^ observed that the heritability estimate for living in deprived neighbourhoods was considerably lower in the twin study (41%) compared to the sibling study (65%), with both twins and siblings being of younger adult age (twins 23−24 years and siblings 31−35 years of age). Whether living in poverty is more strongly influenced by genetic, including epigenetic, factors or by environmental factors and their interplay thus remains to be explored in further studies.

Second, genetic factors contributing to neighbourhood deprivation are not necessarily limited to heritable cognitive capacities. For instance, the genetic correlation of general intelligence and socioeconomic status in the study of Marioni *et al.*^2^ was 0.26, pointing to ‘substantial environmental contributions to socioeconomic status.' Genetic factors predisposing to living in a deprived neighbourhood can also be associated with unequal access to the housing market for phenotypically visible migrants and minorities due to discriminatory practices. We recently observed that, independent of personal income and education, the respective mental health burden was substantially higher in subjects living in socially deprived neighbourhoods;^3^ this effect was substantially stronger in migrants than in non-migrants and (in personal interviews) was mainly attributed to perceived social exclusion and discrimination. In Europe, minorities tend to live in inner cities, and schizophrenia rates are substantially increased in first- and second-generation migrants.^4^ These findings call for a specific exploration of the interaction between genetic factors, social exclusion and schizophrenia manifestation.^5, 6^

As a general remark, we would argue that both supra-additive genetic effects and the presence of complex genotype−phenotype interactions could lead to an overestimation of genetic effects, which could in turn override environmental effects in twin study designs that assume only additive interactions. Moreover, there is emerging evidence that the effects of genetic variation on behavioral phenotypes are amenable to targeted behavioral interventions such as cognitive training,^7^ and such effects may well confound the observed heritability estimates of living in deprived neighbourhoods. These considerations call for study designs that look at complex genotype−phenotype interactions and assess not only genetic but also epigenetic effects.^8^
